# A novel binding site between the voltage-dependent calcium channel Ca_V_1.2 subunit and Ca_V_β2 subunit discovered using a new analysis method for protein–protein interactions

**DOI:** 10.1038/s41598-023-41168-4

**Published:** 2023-08-26

**Authors:** Agnieszka M. Murakami, Katsuhiro Nagatomo, Ichro Miyoshi, Shirou Itagaki, Yasutaka Niwa, Manabu Murakami

**Affiliations:** 1 Department of Pharmacology, Hirosaki University Graduate School of Medicine, 5 Zaifucho, Hirosaki, 036-8562 Japan; 2 Department of Laboratory Animal Medicine, Tohoku University School of Medicine, 2-1 Seiryo-Machi, Aoba-ku, Sendai, 980-8575 Japan; 3 Collaboration Center for Community and Industry, Sapporo Medical University, S1 W17, Chuo-ku, Sapporo, 060-8556 Japan

**Keywords:** Biological techniques, Biotechnology, Physiology

## Abstract

We developed a new method to analyze protein–protein interactions using a dual-inducible prokaryotic expression system. To evaluate protein–protein binding, a chimeric fusion toxin gene was constructed using a DNase-treated short DNA fragment (epitope library) and CcdB, which encodes a DNA topoisomerase II toxin. Protein–protein interactions would affect toxin activity, resulting in colony formation. Using this novel system, we found a new binding site in the voltage-dependent calcium channel α1 subunit (Ca_V_1.2) for the voltage-dependent calcium channel β2 subunit. Prokaryotic expression screening of the β2 subunit using an epitope library of Ca_V_1.2 resulted in two overlapping clones of the C-terminal sequence of Ca_V_1.2. In vitro overlay and immunoprecipitation analyses revealed preferential binding of the C-terminal sequences of Ca_V_1.2 and β2.

## Introduction

Protein–protein interactions are typically studied using biochemical techniques such as crosslinking, coimmunoprecipitation, phage display, and yeast-two hybrid assays. Crosslinking is dependent on chemical reactions in the target protein^1–3^.

Phage display uses bacteriophages to connect genes to a phage coat protein gene, resulting in a phage that displays protein on the outside of bacteria^2^. Phage display requires serial bio-panning (affinity selection) in vitro. Yeast two-hybrid system is dependent on the Gal4 transcriptional activator of *Saccharomyces cerevisiae*^1,3^. Gal4 activates the transcription of a gene involved in galactose utilization. Two-hybrid systems have been used to discover protein–protein interactions^3^. However, yeast two-hybrid screening systems have a high rate of false-positives. Because two-hybrid systems are dependent on transcriptional activity, a means of directly analyzing protein–protein interactions is needed.

We have generated a dual-inducible prokaryotic expression system, pdMAX^4^. pdMAX consists of two inducible expression systems: an arabinose promoter unit and isopropyl-β-d-thiogalactoside (IPTG) inducible unit. This enables two genes to be expressed in one bacterium (*Escherichia coli*). If two molecules interact each other, and one of them has a biological function, this biological function could be influenced by the interaction. Therefore, pdMAX can be used for screening protein–protein interactions.

pdMAX contains *CcdB*, which is a DNA topoisomerase II toxin. With arabinose or IPTG induction, a plasmid without external DNA expresses the *CcdB* toxin, thereby preventing colony formation. In other words, recombinant plasmids with external DNA promote the growth of bacterial colonies. If inserted DNA forms a chimeric protein with *CcdB*, and the chimeric protein retains toxin activity, pdMAX could be used for protein–protein interaction analysis.

In this study, we created an epitope library from a chimera of the mammalian voltage-dependent calcium channel α1 subunit with CcdB under the lac promoter/operator in the pdMAX plasmid. Furthermore, we inserted the β2 subunit gene with the arabinose promoter in the pdMAX system. Using the pdMAX system, we found a novel interaction domain in the α1 subunit.

## Results

### Plasmid construction for protein–protein interaction screening

For protein–protein interaction screening in *E. coli*, we used the pdMAX plasmid, which is a dual prokaryotic expression system^4^.

As a control experiment, a dual expression construct of the full-length Ca_V_β2 subunit gene in the arabinose unit and the Ca_V_1.2-α-interaction domain (AID)-iUnit was prepared (Supplementary Fig. 1). IPTG induction resulted in no colony formation due to chimeric gene of AID and iUnit (CcdB)^5,6^. Induction by arabinose (expression of Ca_**V**_β2 gene) and IPTG (expression of AID-iUnit gene) resulted in colony formation because of an interaction between Ca_V_β2 and AID, which decreased CcdB toxin activity (Supplementary Fig. 1c).

**Figure 1:**
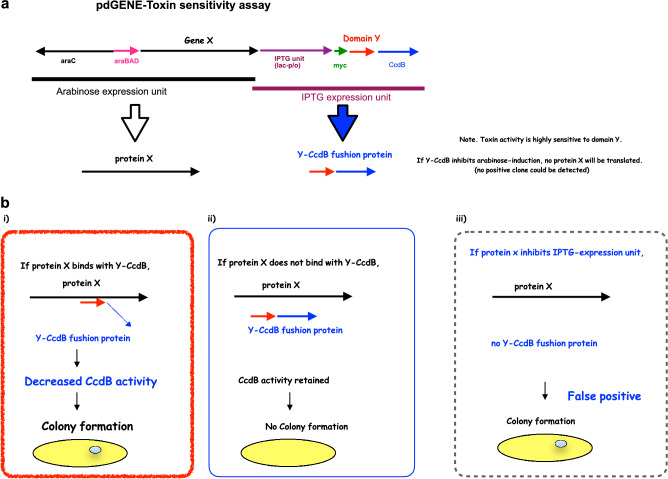
(**a**) Strategy for pdGENE-toxin sensitivity assay using the pdMAX plasmid. The pdMAX system has two functional expression units: arabinose (black line) and IPTG (purple line). Expression of gene X is induced by arabinose and that of the chimeric gene (domain Y and *CcdB*) by IPTG. IPTG sensitivity (*CcdB* toxin induction) should be confirmed. (**b**) Possible results of pdGENE-toxin sensitivity assay. (**i**) If protein X binds with Y-*CcdB*. *CcdB* activity is affected and colonies are formed. (**ii**) If protein X does not bind with Y-*CcdB*. No colonies are formed. (**iii**) If protein X inhibits IPTG expression. Colonies are formed (false-positive clone).

Figure 1a shows a schematic of the epitope library and dual expression system. Full-length cDNA of Gene X is inserted under an arabinose promoter (arabinose unit), which is induced by arabinose. Short fragments of DNA (Domain Y) form chimeric genes with *CcdB* (Y-*CcdB* fusion protein), retaining *CcdB*’s toxic activity. This chimeric gene is induced by IPTG. Because this system is dependent on dual gene expression with the pdMAX plasmid and toxin activity, we named it the pdGENE-Toxin sensitivity assay.

Figure 1b shows possible results of pdMAX epitope expression analysis. If a Y-*CcdB* chimeric protein interacts with protein X, and the interaction affects *CcdB*’s DNA topoisomerase II toxin activity, DNA will be produced and *E. coli* colonies will grow (Fig. 1bi). If protein X does not bind Y-*CcdB*, *CcdB*’s activity is retained and no colonies are formed (Fig. 1bii). If protein X inhibits IPTG induction, no Y-*CcdB* protein is produced and colonies are formed (false-positive clone, Fig. 1biii).

### Construction of pdMAX-expression Ca_V_β2 and Ca_V_1.2-epitope library

Figure 2a shows the strategy to identify β2 subunit interaction domains in the α1 (Ca_V_1.2) subunit. Full-length cDNA of the β2 subunit was inserted into the arabinose unit. Next, short fragments of the α1 gene were inserted to form a chimeric protein with CcdB. If the chimeric product of short Ca_V_1.2 and *CcdB* binds to the Ca_V_β2 subunit, the suppressed toxin activity of the chimeric protein results in colony formation.

**Figure:**
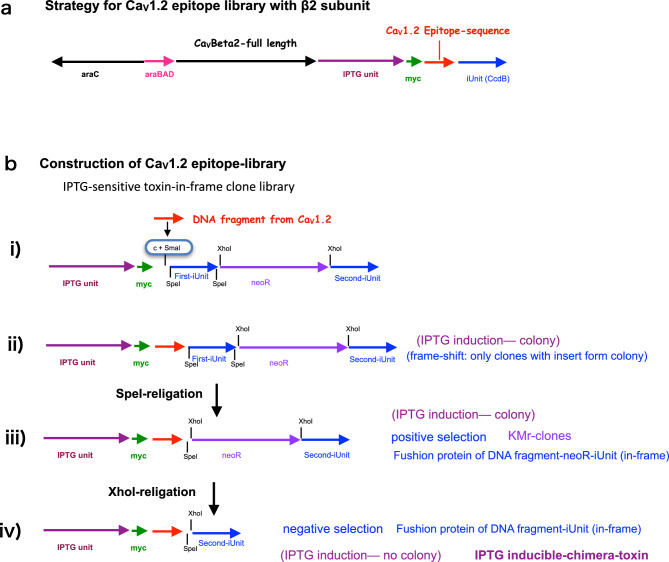
Figure 2 (a) Strategy for creating a Ca_V_1.2 epitope library with the β2 subunit. Full-length cDNA of Ca_V_β2 gene is in the arabinose unit and the Ca_V_1.2 epitope library is in the IPTG unit. (**b**) Construction of a Ca_V_1.2 epitope-library (IPTG-sensitive toxin-in-frame clone library). (**i**) Blunt-ended DNA fragment from Ca_V_1.2 is inserted at the *Sma*I site (with a single frameshift with the first iUnit) of the IPTG unit. (**ii**) Clones with the first iUnit (first library). (**iii**) First iUnit in the library is deleted using *Spe*I and re-ligated. Only clones with the chimeric gene and neomycin-resistance gene form colonies (second library). (**iv**) Neomycin-resistance gene is eliminated using *Xho*I and re-ligated (third library).

### Ca_V_1.2 epitope library

To obtain randomly cleaved Ca_V_1.2 cDNA fragments (GenBank accession number X15539), a partial sequence of Ca_V_1.2 was PCR-amplified using specific primers to prevent amplification of the AID^7–9^, which has high affinity for the β subunit. Next, a Ca_V_1.2 epitope library was prepared using deoxyribonuclease I (DNase I) at 0.25, 0.50 and 1.0 U/ml for 10 min at room temperature (Supplementary Fig. 3a). Randomly shortened fragments of 100–200 bp were pooled. The collected DNA ends were blunted by T4 DNA polymerase. The DNA was inserted into the SmaI site of the IPTG expression unit of pdMAX.

### Construction of the Ca_V_1.2 epitope-library

Figure 2b shows the creation of an IPTG-sensitive *CcdB* in-frame toxin epitope library. To achieve efficient subcloning and construction of in-frame chimeric *CcdB* genes, we prepared the chimeric gene *CcdB* (with an additional cytosine for frameshift)-stop-codon-neomycin-resistance gene-stop codon-second *CcdB* gene in the IPTG unit of pdMAX with *Spe*I to delete the first iUnit, and *Xho*I to delete the neomycin-resistance gene (Fig. 2bi, Supplementary Fig. 2). This expression plasmid enabled subcloning at the *Sma*I site. Only recombinant clones with insert DNA and significantly decreased *CcdB* toxin activity formed colonies with IPTG induction on ampicillin-containing LB agar. The reading frame of the first SpeI recognition site is different in terms of the second SpeI recognition site (frameshift, Supplementary Fig. 2). If both SpeI sites have the same reading frame, chimeric genes of insert DNA with the iUnit should have decreased toxin activity. After ligating the Ca_V_1.2 epitope-library, recombinant constructs were transformed with highly efficient competent cells (Fig. 2bi, Supplementary Fig. 3b panel 1) (XL 10-Gold ultracompetent cells; Agilent Technologies, Santa Clara, CA). Colonies were collected and plasmid DNA was purified (first library).

The first library was digested with *Spe*I, heat-inactivated, and re-ligated. Re-ligated plasmids were transformed and plated on LB agar containing ampicillin, IPTG, and kanamycin (KM) (Fig. 2bii, Supplementary Fig. 3b panel 2). This shifts the reading frame of the chimeric gene because the first and second *Spe*I sites have different reading frames. Recombinant clones grown under this condition express the neomycin-resistance gene upon IPTG induction (second library, positive selection with KM, Fig. 2biii). Using this strategy, only the epitope library, which forms chimeric DNA of Ca_V_1.2 short DNA fragments with the neomycin-resistance gene, and the second iUnit form colonies (Fig. 2biii). After positive selection with kanamycin, the kanamycin-resistance gene was deleted with *Xho*I, heat-inactivated, and re-ligated. Re-ligated plasmids were transformed and plated on LB agar containing ampicillin (Fig. 2biv, third library). Third library plasmid DNA was digested with *EcoR*V at 37 °C for 20 min. Next, *EcoR*V was heat-inactivated at 75 °C for 10 min. EcoRV-digested plasmid DNA was used for full-length Ca_V_β2 insertion.

### Screening of protein–protein interactions

**Figure 3:**
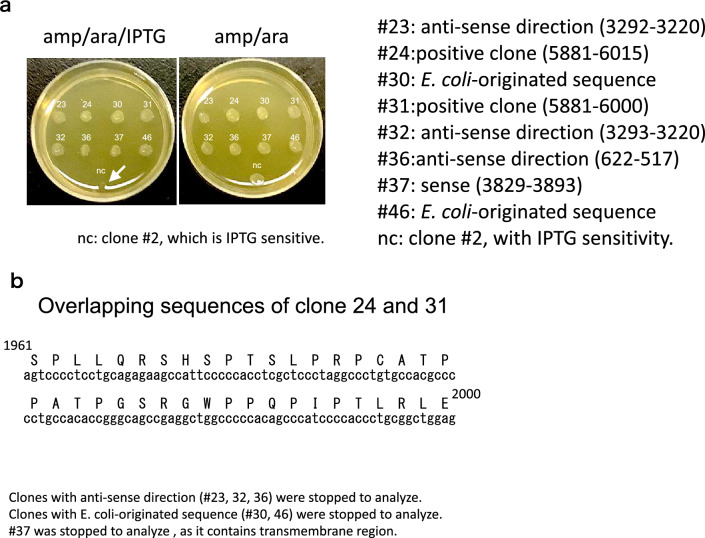
Results of third screening of pdGENE-Toxin sensitivity assay. (**a**) Candidate clones with selection. Selection by ampicillin, arabinose, and IPTG, or ampicillin and arabinose. #23, #24, #30, #31, #32, #36, #37 and #46 form colonies under ampicillin/arabinose/IPTG and ampicillin/arabinose. Negative control was clone #2, which was IPTG-sensitive (negative selection). Sequence direction and corresponding sequences are indicated. Clones in the antisense direction (#23, 32, and 36) and those with an *E. coli*-derived sequence were eliminated. Clone #37 was eliminated because it contained a Ca_V_1.2 transmembrane region. (**b**) Overlapping sequence of clones 24 and 31. Translated amino acid sequences are above nucleotide sequences (single-letter code) and numbered.

After ligation of the Ca_V_β2 gene, colonies were plated on LB agar containing ampicillin, arabinose, and IPTG. After re-plating twice, eight colonies (#23, 24, 30, 31, 32, 36, 37 and 46 from 192 clones) remained and were sequenced (Fig. 3a). Clones with the anti-sense sequence of Ca_V_1.2 (#23, 32, and 36), and those with the *E. coli*-originated sequence (#30 and 46), were eliminated. One clone (#37) had a sequence encoding a transmembrane domain of Ca_V_1.2 and was eliminated. As a result, two clones (#24 and #31, 2 of 192; positivity rate, 1.04%) had overlapping sequences at the C-terminal sequence of Ca_V_1.2 cDNA (Fig. 3b, Supplementary Fig. 4).

### Overlay analysis using the flag-Ca_V_β2 subunit

**Figure 4:**
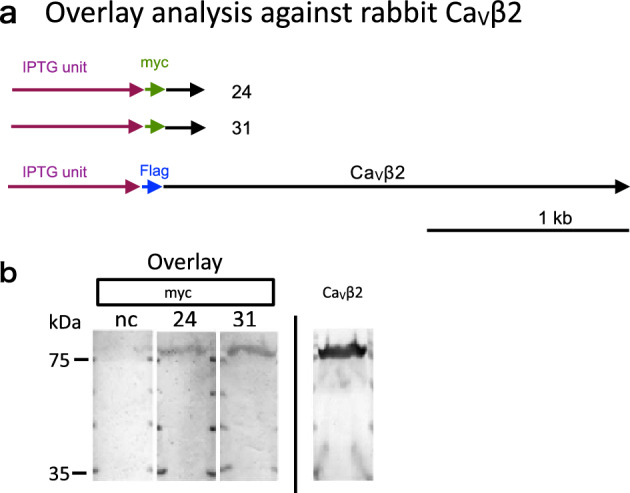
Overlay analysis of #24 and #31 against rabbit Ca_V_β2. (**a**) Expression constructs of #24, #31 and full-length cDNA of Ca_V_β2. Myc gene encoding EQKLISEEDL was tagged to #24 and #31. (**b**) Interaction of #24 and 31 with Ca_V_β2 (Overlay). The anti-Myc antibody revealed a single band that corresponded to the myc-24 or myc-31 fusion protein (75 kDa). The anti-Ca_V_β2 antibody showed an expected single band corresponding to Ca_V_β2. nc, negative control (without the expression protein probe). *E. coli* lysates expressing flag-Ca_V_β2 were separated by SDS-PAGE and transferred to PVDF membranes.

To confirm the interaction between the Ca_V_1.2 (α1C)-derived domain and β2, we conducted an in vitro overlay assay of myc-#24 and myc-#31 clones with the flag-tagged Ca_V_β2 subunit (Fig. 4a). Flag-tagged Ca_V_β2 gene was prepared using the pgMAX plasmid^6^. Flag-tagged Ca_V_β2 protein was separated by 10% sodium dodecyl sulfate–polyacrylamide gel electrophoresis (SDS-PAGE) and transferred to polyvinylidene fluoride (PVDF) membranes.

Two clones (#24 and #31) positive by pdMAX-based screening were incubated in LB medium containing IPTG at 37 °C for 12 h. Cell lysates of myc-#24 and myc-#31 against Flag-tagged Ca_V_β2 were prepared and transferred to PVDF membranes. Clones #24 and #31 interacted with flag-Ca_V_β2 (Fig. 4b) but the negative control (clone #30) did not (Fig. 4b, lane nc). An anti-Ca_V_β2 antibody revealed expression of Ca_V_β2 with the pgMAX/Ca_V_β2 construct (right panel).

### Transient expression in HEK293 cells

**Figure 5:**
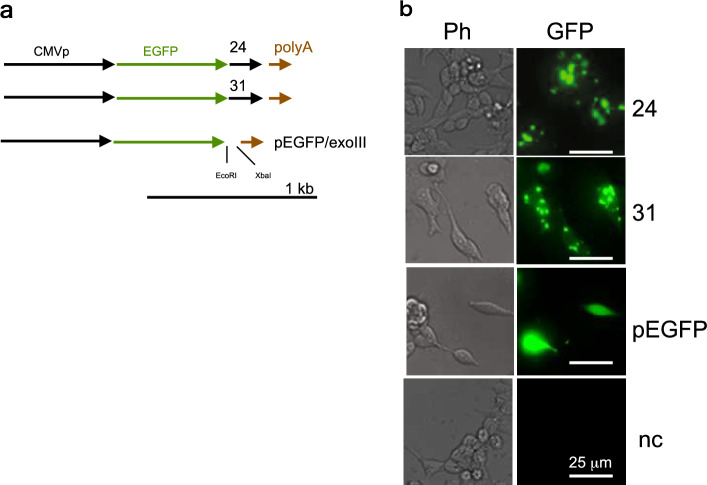
Expression analysis of clones 24 and 31 in HEK-293 cells. (**a**) Expression constructs of clones 24 and 31. Expression constructs were prepared with the CMV promoter, enhanced green fluorescent protein (EGFP), and a poly-A tail. Clones 24 and 31 were tagged with EGFP. (**b**) Expression analysis of EGFP-tagged clones in HEK-293 cells. Constructs were transfected into HEK-293 cells by lipofection. Fluorescence imaging was performed 48 h after transfection. Representative images of EGFP fluorescence (GFP) obtained using a GFP fluorescence filter (505 nm) with blue light (470 nm) and the corresponding phase-contrast (Ph) images are shown. Names of the clones are indicated. Positive control (pEGFP) and negative control (nc; pgMAX without the EGFP gene) are indicated.

Expression constructs were constructed using the pEGFP-C1 vector (Clontech Laboratories, Palo Alto, CA), which has a cytomegalovirus (CMV) promoter and SV40-derived polyA sequence. The candidate sequences (24 and 31) were transferred to *EcoR*I-*Xba*I sites (Fig. 5a). EGFP-#24 and EGFP-#31 constructs showed EGFP clusters (puncta) and faint cytosolic EGFP expression, whereas EGFP-expressing pEGFP-C1 exhibited homogeneous EGFP fluorescence in the cytosol (Fig. 5b).

**Figure 6:**
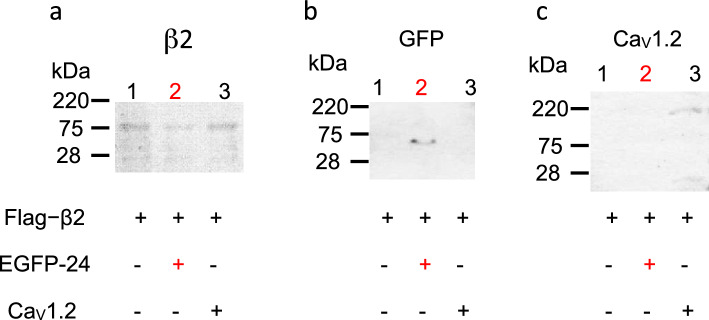
Coimmunoprecipitation analysis of interactions between the β2 subunit and Ca_V_1.2-derived clone (24) in human embryonic kidney 293 (HEK-293) cells. (**a**) Western blot analyses of Ca_V_β2 in HEK-293 T cells. Transected constructs are indicated. Lane, 1 Ca_V_β2; lane 2, Ca_V_β2 and EGFP-24; lane 3, Ca_V_β2 and full-length Ca_V_1.2. (**b**) Coimmunoprecipitation analysis of EGFP-24 and Ca_V_β2. An anti-GFP antibody revealed a single band that corresponded to the EGFP-24 fusion protein (lane 2). (**c**) Coimmunoprecipitation analysis of Ca_V_1.2 and Ca_V_β2. An anti-Ca_V_1.2 antibody revealed a single band (lane 3, 200 kDa).

Immunoprecipitation analysis was performed using transiently expressed flag-Ca_V_β2, GFP-#24, and full-length Ca_V_1.2 cDNA in HEK293T cells (Fig. 6). Expression of the chimeric flag-Ca_V_β2 gene was confirmed (Fig. 6a). Co-expression of EGFP-#24 and flag-Ca_V_β2 resulted in co-precipitation of EGFP-#24 with flag-Ca_V_β2 protein (Fig. 6b, lane 2), indicating a protein–protein interaction. Co-expression of full-length Ca_V_1.2 and flag-Ca_V_β2 resulted in co-precipitation of Ca_V_1.2 with flag-Ca_V_β2 protein (Fig. 6c, lane 3), indicating a protein–protein interaction. Therefore, the #24-coding protein interacted with the Ca_V_β2 subunit.

## Discussion

We established the pdGENE-toxin sensitivity assay to analyze protein–protein interactions using a bacterial-dual expression system.

The pdMAX system does not depend on transcriptional activators, unlike the two-hybrid system^1,3^. Also, this system directly detects protein–protein interactions. Moreover, pdMAX is a prokaryotic system, so there are no nuclear protein–protein interactions. Moreover, the system allows rapid analysis because of the high rate of bacterial growth.

**Figure 7:**
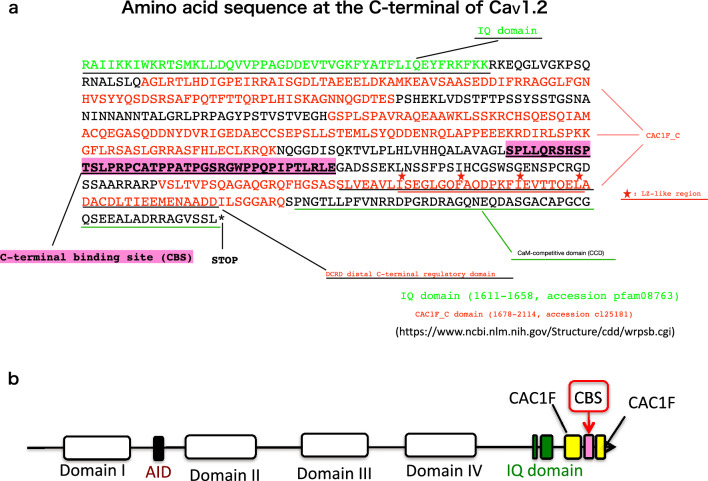
(**a**) Amino acid sequence of the C-terminus of Ca_V_1.2. The IQ domain (light green), CAC1F_C domains (red), C-terminal binding site (CBS), distal C-terminal regulatory domain (black underlined), leucine-zipper-like region (red asterisks), and CaM-competitive domain (green underlined) were obtained from the NCBI database (https://www.ncbi.nlm.nih.gov/Structure/cdd/wrpsb.cgi). (**b**) Schematic of Ca_V_1.2. The four transmembrane domains are labelled I to IV. The high-affinity interaction domain (α-interaction domain; AID) is located between domains I and II. The IQ domain, CAC1F domain, and C-terminal binding site (CBS) are located at the C-terminus.

We found a site of interaction between the Ca_V_1.2 calcium channel α1 subunit and Ca_V_β2 subunit at the carboxy-terminal tail of the α1 subunits. Therefore, we named it the C-terminal Binding Sequence (CBS) (Fig. 7). The sequence is in the CAC1F_C domain (1678–2114, accession cl25181), distal to the isoleucine-glutamine (IQ) motif (1611–1658, accession cl26695). The IQ motif interacts with hydrophobic pockets of Ca^2+^/calmodulin, thereby regulating calcium-dependent inactivation and facilitation. The CAC1F_C domain of Ca_V_1.2 contains a leucine zipper (LZ)-like region distal to the CBS.

The carboxyl-terminal tail of the α1 subunit plays various roles in channel function. In the Ca_V_1.2 subunit, deletion of the distal region of the carboxyl terminus increases the channel opening probability^10^. Moreover, the more proximal EF-hand domain plays a role in Ca^2+^-induced inactivation^11^, whereas the role of the novel CBS site is unclear. Further investigation is needed to clarify the functional importance of the CBS.

**Figure 8:**
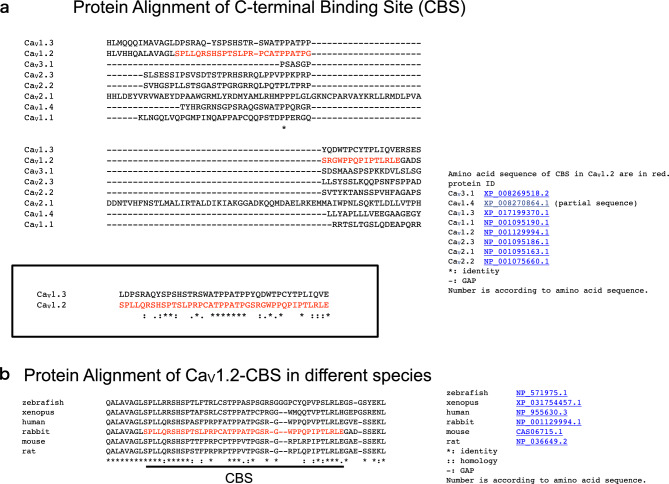
(**a**) Protein alignment of the C-terminal binding sites (CBS) of rabbit α1 subunits. Amino acid sequence alignments of Ca_V_1.1, Ca_V_1.2, Ca_V_1.3, Ca_V_1.4, Ca_V_2.1, Ca_V_2.2, Ca_V_2.3, and Ca_V_3.1 obtained using ClustalW (https://www.genome.jp/tools-bin/clustalw). The CBS in Ca_V_1.2 is in red. Protein IDs are indicated. *, identity; -, gap. Alignment of the CBS of rabbit Ca_V_1.2 and Ca_V_1.3 (inset). CBS sequence of Ca_V_1.3 shows homology with that of Ca_V_1.2. (**b**) Amino acid alignment of the CBS of Ca_V_1.2 in different species. CBS sequences of zebrafish, xenopus, human, rabbit, mouse, and rat were aligned using ClustalW (https://www.genome.jp/tools-bin/clustalw). CBS in rabbit Ca_V_1.2 is in red. Protein IDs are indicated. *, identity; -, gap.

We searched for homologs of the CBS in other voltage-dependent calcium channel α1 subunits (Fig. 8a). Only the CBS of Ca_V_1.3 showed some homology with that of Ca_V_1.2 (Fig. 8a, inset). Ca_V_1.2, Ca_V_1.3, and Ca_V_β2 are expressed in cardiac myocytes^12,13^. Ca_V_1.2 and Ca_V_β2 are expressed in smooth muscle cells^12^. It is possible that binding of the Ca_V_β2 subunit to the CBS influences calcium channel properties. Amino acid sequence alignments of the CBS of Ca_V_1.2 of various species revealed high homology (Fig. 8b).

Walker et al. reported a second Ca_V_β4 subunit interaction domain in the carboxyl-terminal region of Ca_V_2.1 (α1A), which forms P/Q-type voltage-dependent calcium channels^14^. The Walker interaction domain is poorly conserved among α1 isoforms, unlike the novel Ca_V_1.2 interaction domain found in this study. Additionally, alignment of the amino acid sequence of the corresponding amino acid of Ca_V_2.1 indicated no similarity between Ca_V_1.2 and Ca_V_2.1 (Fig. 8a).

Our dual expression system has a number of advantages, such as rapid growth of *E. coli* and dual-inducible expression in a single bacterium. However, a pdGENE-toxin sensitivity assay resulted in a large number of false-positive clones (six of eight clones). Also, only short DNA fragments can be analyzed because the pdGENE-toxin sensitivity assay is dependent on the biological activity of the chimeric toxin. The target genes were eukaryotic (mammalian), but were expressed in the prokaryote *E. coli*.

After the first screening, we isolated 192 independent colonies that were re-plated on a master plate with ampicillin, arabinose, and IPTG; and a replica plate with ampicillin and IPTG. We compared the growth of all clones and further re-plated arabinose- (positively selected) and IPTG (negatively selected)-sensitive clones. After this second screening, we sequenced eight clones. It is possible that only two of these eight clones were true positives (positivity rate, 1.04%). We prepared a chimera comprising a part of the Ca_V_1.2 gene and the *CcdB* toxin gene. If the binding domain of Ca_V_β2 formed a chimeric toxin, the number of false-positive clones would be smaller. Therefore, two different toxin genes could be used to decrease the number of false-positive clones.

Our novel bacterial dual expression system has utility for various types of research, such as expression analysis and the design of therapeutic peptides (or nanobody-like heavy chain antibodies) that interact with bacterial or viral proteins.

In conclusion, we established a novel system to analyze protein–protein interactions. Using this system, we found a Ca_V_β2 interaction site in the C-terminus of Ca_V_1.2.

## Methods

### Experimental procedures

#### Plasmid construction

For screening, we used the pdMAX plasmid^4^. Plasmids were constructed with a rapid method (Murakami-system)^15^. pdMAX has two inducible prokaryotic promoters, arabinose and lac, on one plasmid in *Escherichia coli*. The pdMA system has two inducible promoters: P_BAD_ for arabinose and lac for IPTG. The pdMAX system enables the induction and analysis of the expression of two different genes.

Figure 1a shows a schematic of the epitope library and dual expression system. Full-length cDNA of Gene X is inserted under the arabinose promoter, which is induced by arabinose. Short-fragment DNA (Domain Y) forms a chimeric gene with *CcdB* (Y-*CcdB* fusion protein), retaining *CcdB*’s toxic activity. This chimeric gene is induced by IPTG.

Figure 1b shows the possible results of pdMAX epitope expression analysis. If a Y-*CcdB* chimeric protein interacts with protein X, and the interaction affects *CcdB*’s DNA topoisomerase II toxin activity, DNA is produced and *E. coli* colonies grow (Fig. 1bi). If protein X does not bind with Y-*CcdB*, *CcdB*’s activity is retained and no colonies will grow (Fig. 1bii). If protein X inhibits IPTG induction, no Y-*CcdB* protein is expressed and colonies form (Fig. 1biii).

Figure 2a shows the strategy used to discover β2 subunit interaction domains in the α1 (Ca_V_1.2) subunit. Full-length cDNA of the β2 subunit gene was inserted into the arabinose unit. Short fragments of the α1 gene were inserted and formed a chimeric protein with the *CcdB* gene product.

#### Ca_V_1.2 epitope library

To obtain randomly cleaved Ca_V_1.2 cDNA fragments (GenBank ac- cession number X15539), a partial sequence of Ca_V_1.2 (5103 bp) was PCR-amplified with specific primers (rabbit Ca_V_1.2AIDfor, 5′-ACTCAGGCAGAAGACATCGACCCT-3’ and rabbit Ca_V_rev, 5’- CTACAGGCTGCTGACGCCGGCCCT-3′). Because rabbit Ca_V_1.2AIDfor is a specific primer that codes just after the alpha-binding domain (AID) sequence, the amplicon does not include an AID-coding sequence. Next, 10 μg of amplicon was incubated with 50 mM Tris–HCl (pH 7.5), 50 μg/ml bovine serum albumin (BSA), and 10 mM MnCl2 and DNase I (0.25, 0.50 and 1.0 U/mL) for 10 min at room temperature. Under these conditions, cleavage is random and fragment sizes can be controlled by varying the enzyme concentration^16^. The reactions were stopped by adding EDTA (pH 8.0) and glycerol at 16.7 mM and 5% (v/v), respectively. Aliquots of the reaction mixtures were analyzed by agarose gel electrophoresis (Supplementary Fig. 3a). Reaction mixtures containing fragments 100–200 bp in length were pooled, loaded onto a 2% agarose gel, and electrophoresed. Next, the DNA fragments were purified. Assuming an average size of 135 bp, 22.5 pmol DNA ends were blunted by T4 DNA polymerase (1 U) in the presence of dNTPs (0.1 mM each of dCTP, dGTP, and dTTP, and 1 mM of dATP) for 20 min at 15 °C. After inactivation of the enzyme (10 min, 75 °C), DNA was inserted into the *Sma*I site of pdMAX (IPTG expression unit).

Figure 2b shows the establishment of an IPTG-sensitive *CcdB* gene in-frame toxin epitope library.

For subcloning, we prepared a chimeric gene of first *CcdB* (first iUnit, with an additional cytosine for frameshift)-neomycin-resistance gene-second *CcdB* gene (second iUnit) in the IPTG unit of pdMAX with *Spe*I to delete the first iUnit and *Xho*I to delete the neomycin-resistance gene (Fig. 2bi).

This expression plasmid enabled efficient subcloning at the *Sma*I site (only recombinant clones with insert DNA and significantly decreased *CcdB* toxin activity of the first iUnit formed colonies) with IPTG induction on ampicillin-containing LB agar.

Colonies were collected and plasmid DNA was purified (first library). The first library was digested with *Spe*I at 37 °C for 2 h (Fig. 2Bii). *Spe*I was heat-inactivated at 75 °C for 10 min followed by cooling at 4 °C. One microgram of digested plasmid DNA was incubated with DNA ligase at 16 °C for 30 min. Ligated plasmids were transformed and incubated at 37 °C for 1 h in LB medium containing IPTG for induction of the neomycin-resistance gene, and plated on LB agar containing ampicillin, IPTG, and KM. Recombinant clones grown under these conditions expressed the neomycin-resistance gene with IPTG induction (second library, positive selection with KM, Fig. 2Biii). Using this strategy, only epitope libraries, which that form chimeric DNA of Ca_V_1.2 short DNA fragment with the neomycin-resistance gene, and the second iUnit form colonies (Supplementary Figs. 1b, and 3b, panel 2).

Colonies were collected and plasmid DNA was purified. Purified plasmid DNA (second library) was digested with *Xho*I at 37 °C for 2 h (Fig. 2bii). *Xho*I was heat-inactivated at 75 °C for 10 min, followed by cooling at 4 °C. One microgram of digested plasmid DNA was incubated with DNA ligase at 16 °C for 30 min. Ligated plasmids were transformed and incubated at 37 °C for 1 h in LB medium, and plated on LB agar containing ampicillin. Colonies were collected and plasmid DNA was purified (third library, Fig. 2Biv). Because the neomycin-resistance gene is in the same frame as the second iUnit, the artificial gene of the Ca_V_1.2 DNA fragment, i.e., neomycin-resistance gene-second iUnit, has the same codon frame. Third library plasmid DNA was digested with *EcoR*V at 37 °C for 20 min. EcoRV was heat-inactivated at 75 °C for 10 min. *EcoR*V-digested plasmid DNA was used for full-length Ca_V_β2 insertion. Full-length Ca_V_β2 (XM_017347581.2) cDNA (1827 bp) was amplified with pfu DNA polymerase and specific oligo DNA primers (rabbit Beta2for, 5′-ATGAACCAGGCGAGTGGACTGGAC-3′ and rabbit Beta3rev, 5′-TCATTGGCGGATGTAAACATCCCT-3′; Agilent Technologies). The conditions for PCR using high-fidelity Pfu DNA polymerase were empirically modified to 25 cycles of denaturation at 98 °C for 10 s, annealing at the calculated temperature [ca. 50 °C] for 30 s, and extension at 72 °C for 120 s. The PCR products were purified using a Gel Extraction Kit (Macherey–Nagel GmbH, Duren, Germany). 

After ligating full-length Ca_V_β2 cDNA, recombinant constructs were transformed with highly efficient competent cells (XL 10-Gold ultracompetent cells; Agilent Technologies). Next, *E. coli* cells were incubated at 37 °C for 1 h in LB medium and plated on LB agar containing ampicillin, arabinose, and IPTG at 37 °C for 16 h. Colonies were re-plated on a master plate with ampicillin, arabinose, and IPTG; a replica plate contained ampicillin and IPTG. After two serial re-plating, eight colonies (#23, 24, 30, 31, 32, 36, 37 and 46) were sequenced (Fig. 3a). Clones with the antisense sequence of Ca_V_1.2 (#23, 32, and 36) and those with an *E. coli*-derived sequence (#30 and 46), were eliminated. One clone (#37) had a sequence encoding a transmembrane domain of Ca_V_1.2 and was eliminated. The remaining two clones (#24 and 31) had overlapping sequences at the C-terminal sequence of Ca_V_1.2 cDNA (Fig. 3b).

#### Overlay analysis

Two clones (#24 and 31) were analyzed using the overlay method.

Full-length cDNA of Ca_V_β2 was prepared as reported previously (Fig. 4a)^6^. We prepared the pgMAX expression plasmid with a flag-tag sequence containing the full-length cDNA of Ca_V_β2. Flag-Ca_V_β2 chimera gene expression was induced by IPTG. *E. coli* clones were incubated in LB medium containing ampicillin and IPTG at 37°C for 12 h. Cells were collected from 1.0 mL of bacterial culture and centrifuged at 6000×*g* for 1 min. Cells were resuspended in 0.2 mL of CelLytic B reagent for 10 min according to the manufacturer’s protocol (Sigma-Aldrich, St. Louis, MO). Cell lysate containing Flag-Ca_V_β2 chimera protein was centrifuged at 6000×*g* for 5 min to pellet insoluble material. Five percent of the supernatant was resolved by 10% SDS-PAGE. Recombinant Flag-Ca_V_β2 chimeric protein was separated by SDS-PAGE. The proteins were transferred to PVDF membranes.

For overlay analysis, candidate clones (#24 and 31) with a myc-tag sequence were subjected to IPTG-protein induction in LB medium containing ampicillin and IPTG at 37 °C for 12 h. Cells were collected from 4.0 mL of bacterial culture and centrifuged at 6000×*g* for 1 min. The cells were resuspended in 1.0 mL of lysis buffer (1 mM EDTA, 1 mg/mL lysozyme) and incubated at room temperature for 15 min. Cell lysate containing each clone was centrifuged at 6000×*g* for 5 min to pellet insoluble material. Ten percent of the supernatant was used for overlay analysis.

For screening using the myc-24 or 31 constructs, PVDF membranes containing Flag-Ca_V_β2 chimera protein were blocked in TBST (150 mM NaCl, 20 mM Tris–HCl [pH 7.5], 0.05% Tween-20) with 0.1% BSA for 1 h, followed by incubation (16 h at 4 °C) with 10% cell lysate (myc-24 or myc-31) in TBST containing 5% (w/v) BSA and complete TM protease inhibitor cocktail (Roche, Basel, Switzerland). The membranes were washed three times with TBST containing 0.1% (v/v) Tween-20. Next, a monoclonal antibody specific for myc-tag (Medical & Biological Laboratories Co. Ltd., Tokyo, Japan) was incubated in TBST containing 1% (w/v) BSA at 4 °C for 12 h. The membranes were washed three times with TBST containing 0.1% (v/ v) Tween-20. An anti-mouse IgG alkaline phosphatase conjugate (Promega, Madison, WI) was incubated in TBST containing 1% (w/v) BSA at 25 °C for 1 h. Next, membranes were washed three times with TBST containing 0.1% (v/ v) Tween-20 and three times with TBS. Subsequently, myc-tag proteins were visualized with Western Blue (Promega). To confirm flag-Ca_V_β2 chimeric protein expression, an antibody specific for flag protein (Sigma-Aldrich) was used for immunodetection.

Commercially available monoclonal antibodies specific for myc (Medical & Biological Laboratories Co. Ltd.), flag (Sigma-Aldrich), and anti-mouse IgG alkaline phosphatase conjugate (Promega) were used for immunodetection.

### Transient expression in HEK293 cells

#### Mammalian expression construct

Expression constructs were generated using the pEGFP-C1 vector (Clontech Laboratories). Candidate sequences (24 and 31) were transferred to *EcoR*I-*Xba*I sites (Fig. 5a).

#### Cell culture and transfection

Cell culture and lipofection were performed as described previously^9^. Human embryonic kidney cells (HEK293; ATCC CRL 1573) were cultured in Dulbecco’s modified Eagle’s medium supplemented with 10% fetal bovine serum. Exponentially growing cells were plated onto 35-mm dishes and lipofection was performed using commercially prepared lipofectamine (Invitrogen, Carlsbad, CA). The mammalian expression constructs were transfected. After 48 h, EGFP fluorescence was visualized.

#### Immunoprecipitation of EGFP-24 with the flag-Ca_V_β2 subunit in HEK293 cells

Immunoprecipitation was performed using a Protein G Immunoprecipitation Kit (Sigma-Aldrich)^6^. Cell pellet was resuspended in 1.0 mL of lysis buffer (20 mM sodium phosphate, 150 mM sodium chloride, 10% glycerol, 1 mM ethylenediaminetetraacetic acid, 0.5% Triton-X 100 [pH 7.2]) and complete TM protease inhibitor cocktail (Roche, Basel, Switzerland) at ∼ 1 mg/mL. The suspension was placed on ice for 1 h and centrifuged at 10,000×*g* at 4 °C for 15 min. The cleared lysate was incubated at 4 °C for 1 h with 2 μg of a monoclonal anti-flag antibody (Sigma-Aldrich) against flag-tagged Ca_V_β2 protein. Protein G Sepharose (50 μL) was added to the samples, which were incubated for 16 h at 4 °C. The immunoprecipitate was washed five times with 1 mL of immunoprecipitation buffer before being eluted with 60 μL of Laemmli buffer. The eluted product (15 μL) and an equal volume of whole-cell lysate were subjected to 10% SDS-PAGE and Western blotting (ProBlot II AP; Promega) with an anti-β2 (Sigma-Aldrich), anti-GFP (GeneTex Inc., Irvine, CA), or anti-Ca_V_1.2 (Alomone, Jerusalem, Israel) polyclonal antibody, which recognizes the intracellular loop between domains II and III of Ca_V_1.2. Anti-rabbit IgG alkaline phosphatase conjugate (Promega) was used for immunodetection.

### Supplementary Information

Supplementary Figures.

## Data Availability

All data are in the manuscript or supporting information.
