# Supplementary material for: A novel binding site between the voltage-dependent calcium channel CaV1.2 subunit and CaVβ2 subunit discovered using a new analysis method for protein–protein interactions
Source: Sci Rep. 2023 Aug 26;13:13986. doi: 10.1038/s41598-023-41168-4 (PMC10460381; doi:10.1038/s41598-023-41168-4)
Supplement: Supplementary file 1 — Supplementary Figures. [file 41598_2023_41168_MOESM1_ESM.pdf]

a Control experiment with full-length Ca$_V$β2 and AID

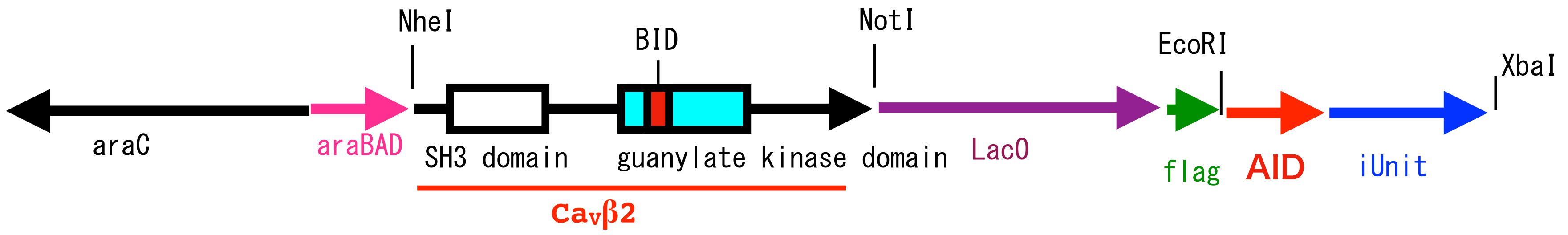

SH3 and guanylate kinase domains, which contain the β-binding domain (BID) of Ca$_V$β2, are indicated. Rabbit AID of Ca$_V$1.2 is indicated. Restriction enzyme sites (*Nhe*I, *Not*I, *EcoR*I and *Xba*I) are indicated.

b Artificial chimeric gene (flag-AID-iUnit)

flag-tag

```
 M  T  M  G  S  I  T  P  S  Y  L  G  D  T  I  E  D  Y  K  D  D  D  D  K  G  S  M  N  S  Q
ATGACCATGGGATCCATTACGCCAAGCTATTTAGGTGACACTATAGAAGACTACAAAGACGATGACGACAAGGGATCCATGAATTCACAG

 Q  L  E  E  D  L  K  G  Y  L  D  W  I  T  Q  A  E  I  G  P  Y  V  R  Q  F  K  V  Y  T  Y
CAGCTGGAAGAGGACCTCAAAGGCTACCTGGACTGGATCACTCAGGCAGAAATCGGGCCCTACGTACGGCAGTTTAAGGTTTACACCTAT

 K  R  E  S  R  Y  R  L  F  V  D  V  Q  S  D  I  I  D  T  P  G  R  R  M  V  I  P  L  A  S
AAAAGAGAGAGCCGTTATCGTCTGTTTGTGGATGTACAGAGTGATATTATTGACACGCCGGGGCGACGGATGGTGATCCCCCTGGCCAGT

 A  R  L  L  S  D  K  V  S  R  E  L  Y  P  V  V  H  I  G  D  E  S  W  R  M  M  T  T  D  M
GCACGTCTGCTGTCAGATAAAGTCTCCCGTGAACTTTACCCGGTGGTGCATATCGGGGATGAAAGCTGGCGCATGATGACCACCGATATG

 A  S  V  P  V  S  V  I  G  E  E  V  A  D  L  S  H  R  E  N  D  I  K  N  A  I  N  L  M  F
GCCAGTGTGCCGGTCTCCGTTATCGGGGAAGAAGTGGCTGATCTCAGCCACCGCGAAAATGACATCAAAAACGCCATTAACCTGATGTTC

 W  G  I  *
TGGGGAATATAA
```

AID

iUnit

Nucleotide sequence and corresponding amino acid sequences are shown. The translated amino acid sequence is shown above the nucleotide sequence (single-letter code). Flag tag (green), AID (red), and iUnit (*CcdB*, blue) sequences are shown.

c Induction of flag-AID-iUnit gene

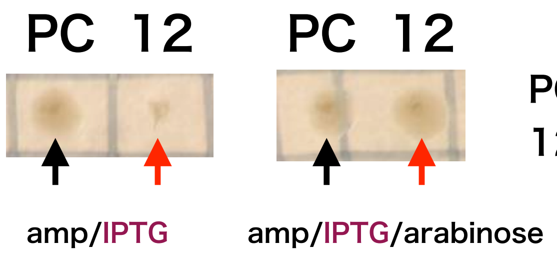

PC: positive control
12: Cav1.2AID

Ampicillin and IPTG containing agar induced flag-AID-iUnit, resulting in no colony formation (left panel, red arrow). Ampicillin, IPTG, and arabinose-containing agar resulted in colony formation (right panel, red arrow). Positive control (PC, pBluescript containing *E. coli*). Plasmid with full-length Ca$_V$β2 and Ca$_V$1.2AID.

Supplementary Information Figure 1

Nucleotide sequence of the IPTG unit

myc-tag

```
 M  T  M  G  S  E  Q  K  L  I  S  E  E  D  L  M  N  S  G  G  G  G  T  S  P  G  L  V  Y  V
atgaccatgGGATCCgaacaaaaactcatctcagaagaggatctgATGAATTCaGGCGGCGGAGGGACGTCCCCGGGACTAGTcTacgta

 R  Q  F  K  V  Y  T  Y  K  R  E  S  R  Y  R  L  F  V  D  V  Q  S  D  I  I  D  T  P  G  R
cggcagtttaaggtttacacctataaaagagagagccgttatcgtctgtttgtggatgtacagagtgatattattgacacgccggggcga

 R  M  V  I  P  L  A  S  A  R  L  L  S  D  K  V  S  R  E  L  Y  P  V  V  H  I  G  D  E  S
cggatggtgatcccctggccagtgcacgtctgctgtcagataaagtctcccgtgaactttacccggtggtgcatatcggggatgaaagc

 W  R  M  M  T  T  D  M  A  S  V  P  V  S  V  I  G  E  E  V  A  D  L  S  H  R  E  N  D  I
tggcgcatgatgaccaccgatatggccagtgtgccggtctccgttatcggggaagaagtggctgatctcagccaccgcgaaaatgacatc

 K  N  A  I  N  L  M  F  W  G  I  *  T  S  V  G  V  G  V  D  F  L  E  [illegible]  E  Q  D  G  L  H
aaaaacgccattaacctgatgttctggggaatataaACTAGTGTCGGGGTCGGGGTCGACttCTCGAGAttgaacaagatggattgcac

 A  G  S  P  A  A  W  V  E  R  L  F  G  Y  D  W  A  Q  Q  T  I  G  C  S  D  A  A  V  F  R
gcaggttctccggccgcttgggtggagaggctattcggctatgactgggcacaacagacaatcggctgctctgatgccgccgtgttccgg

 L  S  A  Q  G  R  P  V  L  F  V  K  T  D  L  S  G  A  L  N  E  L  Q  D  E  A  A  R  L  S
ctgtcagcgcaggggcgcccggttctttttgtcaagaccgacctgtccggtgccctgaatgaactgcaagacgaggcagcgcggctatcg

 W  L  A  T  T  G  V  P  C  A  A  V  L  D  V  V  T  E  A  G  R  D  W  L  L  L  G  E  V  P
tggctggccacgacgggcgttccttgcgcagctgtgctcgacgttgtcactgaagcgggaagggactggctgctattgggcgaagtgccg

 G  Q  D  L  L  S  S  H  L  A  P  A  E  K  V  S  I  M  A  D  A  M  R  R  L  H  T  L  D  P
gggcaggatctcctgtcatctcaccttgctcctgccgagaaagtatccatcatggctgatgcaatgcggcggctgcatacgcttgatccg

 A  T  C  P  F  D  H  Q  A  K  H  R  I  E  R  A  R  T  R  M  E  A  G  L  V  D  Q  D  D  L
gctacctgcccattcgaccaccaagcgaaacatcgcatcgagcgagcacgtactcggatggaagccggtcttgtcgatcaggatgatctg

 D  E  E  H  Q  G  L  A  P  A  E  L  F  A  R  L  K  A  S  M  P  D  G  E  D  L  V  V  T  H
gacgaagagcatcaggggctcgcgccagccgaactgttcgccaggctcaaggcgagcatgcccgacggcgaggatctcgtcgtgacccat

 G  D  A  C  L  P  N  I  M  V  E  N  G  R  F  S  G  F  I  D  C  G  R  L  G  V  A  D  R  Y
ggcgatgcctgcttgccgaatatcatggtggaaaatggccgcttttctggattcatcgactgtggccggctgggtgtggcggaccgctat

 Q  D  I  A  L  A  T  R  D  I  A  E  E  L  G  G  E  W  A  D  R  F  L  V  L  Y  G  I  A  A
caggacatagcgttggctacccgtgatattgctgaagagcttggcggcgaatgggctgaccgcttcctcgtgctttacggtatcgccgct

 P  D  S  Q  R  I  A  F  Y  R  L  L  D  E  F  F  *  L  E  G  I  G  P  Y  V  R  Q  F  K  V
cccgattcgcagcgcatcgccttctatcgccttcttgacgagttcttctgaCTCGAGGGATCGGGCCCTacgtacggcagtttaaggtt

 Y  T  Y  K  R  E  S  R  Y  R  L  F  V  D  V  Q  S  D  I  I  D  T  P  G  R  R  M  V  I  P
tacacctataaaagagagagccgttatcgtctgtttgtggatgtacagagtgatattattgacacgccggggcgacggatggtgatcccc

 L  A  S  A  R  L  L  S  D  K  V  S  R  E  L  Y  P  V  V  H  I  G  D  E  S  W  R  M  M  T
ctggccagtgcacgtctgctgtcagataaagtctcccgtgaactttacccggtggtgcatatcggggatgaaagctggcgcatgatgacc

 T  D  M  A  S  V  P  V  S  V  I  G  E  E  V  A  D  L  S  H  R  E  N  D  I  K  N  A  I  N
accgatatggccagtgtgccggtctccgttatcggggaagaagtggctgatctcagccaccgcgaaaatgacatcaaaaacgccattaac

 L  M  F  W  G  I  *
ctgatgttctggggaatataa
```

First-iUnit

neomycin resistant gene

Second-iUnit

SmaI: CCCGGG

SpeI: ACTAGT

XhoI: CTCGAG

Nucleotide sequences and corresponding amino acid sequences of IPTG units are shown. Translated amino acid sequences are given above the nucleotide sequences (single-letter code). Myc tag gene (EQKLISEEDL, green), first iUnit (*CcdB*, blue), neomycin-resistance gene (purple), and second iUnit (blue) sequences are shown. Recognition sites of *Sma*I (CCC′GGG), *Spe*I (A′CTAGT), and *Xho*I (C′TCGAG) are indicated.

Supplementary Information Figure 2

Construction of the $Ca_V1.2$ epitope library

a DNase I digestion of $Ca_V1.2$

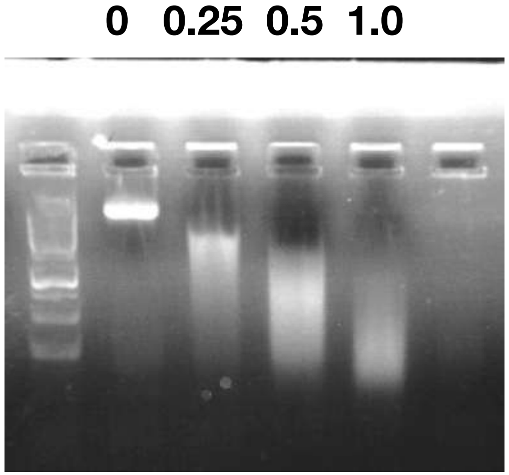

b First library (1), *Spe*I-digested-re-ligated second library (2) and its negative control (NC)

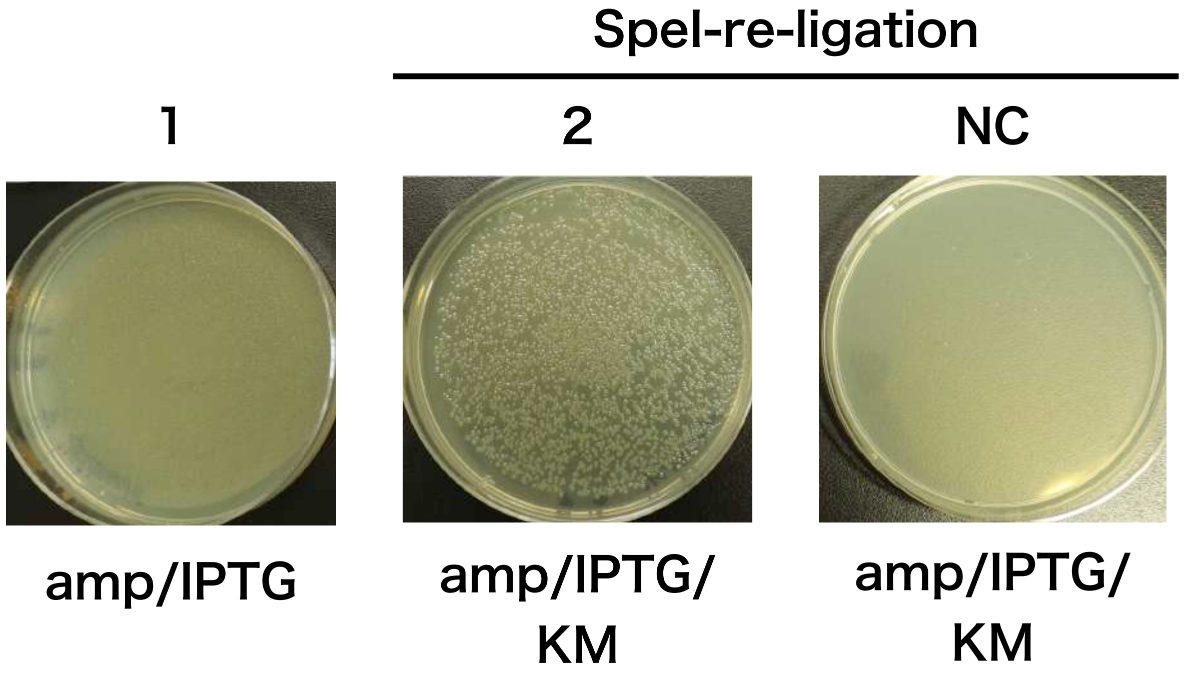

a. DNase I digestion of $Ca_V1.2$

PCR-amplified $Ca_V1.2$ sequence was partially digested with DNase I at the indicated concentrations.

b. First library (1), *Spe*I-digested-re-ligated second library (2) and its negative control

As the negative control, ptMAX was digested with SpeI and re-ligated. No colonies were formed (NC). Agar compositions (ampicillin, IPTG, and kanamycin [KM]) are indicated.

Supplementary Information Figure 3

Nucleotide sequences of clone 24 and 31

#24 Ca_V1.2 (5881-6015)

```
 S  P  L  L  Q  R  S  H  S  P  T  S  L  P  R  P  C  A  T  P
agtcccctcctgcagagaagccattcccccacctcgctccctaggccctgtgccacgccc

 P  A  T  P  G  S  R  G  W  P  P  Q  P  I  P  T  L  R  L  E
cctgccacaccgggcagccgaggctggcccccacagcccatccccaccctgcggctggag

 G  A  D  S  S
ggggccgactccagt
```

#31 Ca_V1.2 (5881-6000)

```
 S  P  L  L  Q  R  S  H  S  P  T  S  L  P  R  P  C  A  T  P
agtcccctcctgcagagaagccattcccccacctcgctccctaggccctgtgccacgccc

 P  A  T  P  G  S  R  G  W  P  P  Q  P  I  P  T  L  R  L  E
cctgccacaccgggcagccgaggctggcccccacagcccatccccaccctgcggctggag
```

Translated amino acid sequences are given above the nucleotide sequences (single-letter code). The numbers of amino acids in Ca_V1.2 of #24 and #31 are indicated.

Supplementary Information Figure 4

Original pictures of Figure 4 (A) and 6 (B)

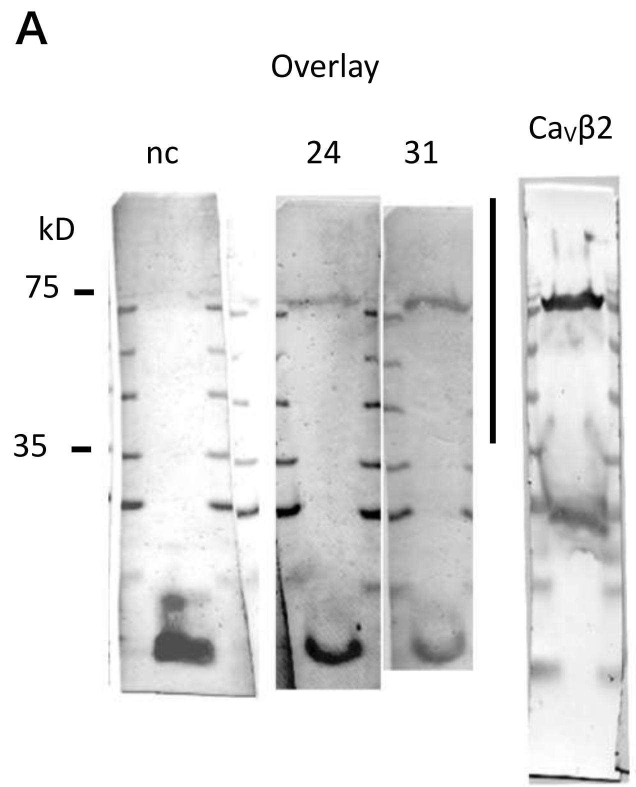

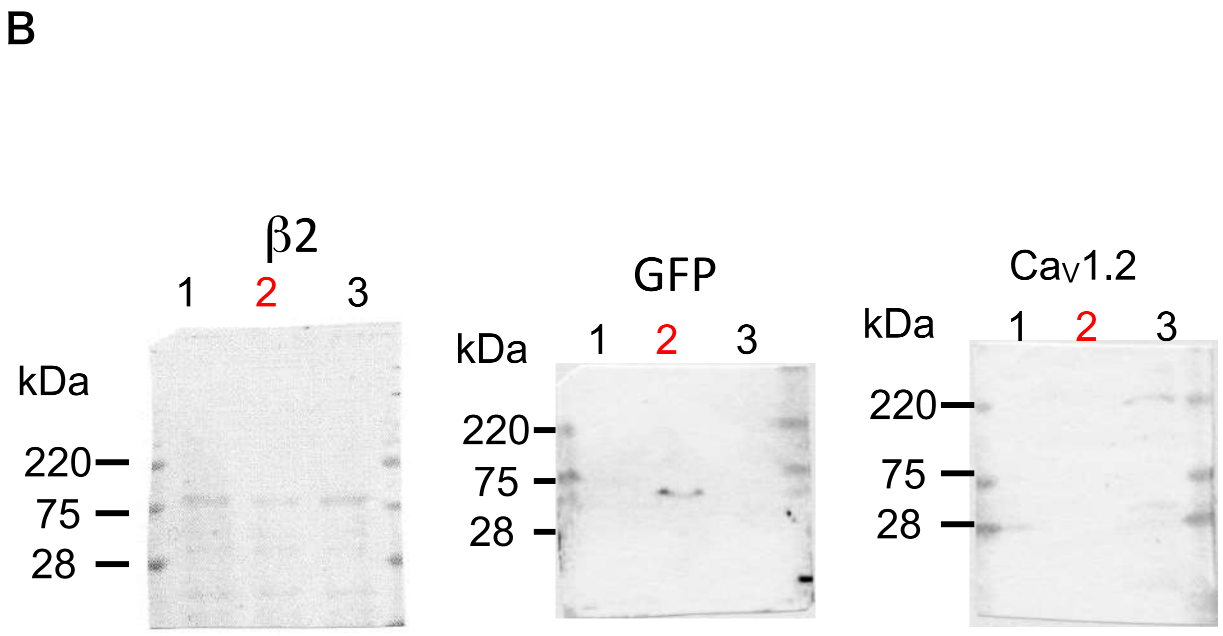

Supplementary Information Figure 5

Nucleotide sequence of ptMAX

ATCGATGCATAATGTGCCTGTCAAATGGACGAAGCAGGGATTCTGCAAACCCTATGCTACTCCGTCAAGCCGTCAATTGTCTGATTCGTTACCAATTATGACAACTTGACGGCTACATCATTCACTTTTTCTTCACAACCGGCACGGAACTCGCTCGGGCTGGCCCCGGTGCATTTTTTAAATACCC
GCGAGAAATAGAGTTGATCGTCAAAACCAACATTGCGACCGACGGTGGCGATAGGCATCCGGGTGGTGCTCAAAAGCAGCTTCGCCTGGCTGATACGTTGGTCCTCGCGCCAGCTTAAGACGCTAATCCCTAACTGCTGGCGGAAAAGATGTGACAGACGCGACGGCGACAAGCAAACATGCT
GTGCGACGCTGGCGATGTCAAAATTGCTGTCTGCCAGGTGATCGCTGATGTACTGACAAGCCTCGCGTACCCGATTATCCATCGGTGGATGGAGCGACTCGTTAATCGCTTCCATGCGCCGCAGTAACAATTGCTCAAGCAGATTTATCGCCAGCAGCTCCGAATAGCGCCCTTCCCCTTGCCCGG
CGTTAATGATTTGCCCAAACAGGTCGCTGAAATGCGGCTGGTGCGCTTCATCCGGGCGAAAGAACCCCGTATTGGCAAATATTGACGGCCAGTTAAGCCATTCATGCCAGTAGGCGCGCGGACGAAAGTAAACCCACTGGTGATACCATTCGCGAGCCTCCGGATGACGACCGTAGTGATGAATC
TCTCCTGGCGGGAACAGCAAAATATCACCCGGTCGGCAAACAAATTCTCGTCCCTGATTTTTCACCACCCCCTGACCGCGAATGGTGAGATTGAGAATATAACCTTTCATTCCCAGCGGTCGGTCGATAAAAAAATCGAGATAACCGTTGGCCTCAATCGGCGTTAAACCCGCCACCAGATGGGC
ATTAAACGAGTATCCCGGCAGCAGGGGATCATTTTGCGCTTCAGCCATACTTTTCATACTCCCGCCATTCAGAGAAGAAACCAATTGTCCATATTGCATCAGACATTGCCGTCACTGCGTCTTTTACTGGCTCTTCTCGCTAACCAAACCGGTAACCCCGCTTATTAAAAGCATTCTGTAACAAAGC
GGGACCAAAGCCATGACAAAAACGCGTAACAAAAGTGTCTATAATCACGGCAGAAAAGTCCACATTGATTATTTGCACGGCGTCACACTTTGCTATGCCATAGCATTTTTATCCATAAGATTAGCGGATCCTACCTGACGCTTTTTATCGCAACTCTCTACTGTTTCTCCATACCCGTTTTTTTGGGC
TAGAAATAATTTTGTTTAACTTTAAGAAGGAGATATACATATGGCTAGCGAGCTCAAGCTTGATATCAGATCTGGTACCTACGTACGGCAGTTTAAGGTTTACACCTATAAAAGAGAGAGCCGTTATCGTCTGTTTGTGGATGTACAGAGTGATATTATTGACACGCCGGGGCGACGGATGGTGATCCC
CCTGGCCAGTGCACGTCTGCTGTCAGATAAAGTCTCCCGTGAACTTTACCCGGTGGTGCATATCGGGGATGAAAGCTGGCGCATGATGACCACCGATATGGCCAGTGTGCCGGTCTCCGTTATCGGGGAAGAAGTGGCTGATCTCAGCCACCGCGAAAATGACATCAAAAACGCCATTAACCTGA
TGTTCTGGGGAATATAAAGCGGCCGCTTTTACGGTTCCTGGGCTTTTGCTGGCCTTTTGCTCACATGTTCTTTCCTGCGTTATCCCCTGATTCTGTGGATAACCGTATTACCGCCTTTGAGTGAGCTGATACCGCTCGCCGCAGCCGAACGACCGAGCGCAGCGAGTCAGTGAGCGAGGAAGCGGA
AGAGCGCCCAATACGCAAACCGCCTCTCCCCGCGCGTTGGCCGATTCATTAATGCAGCTGGCACGACAGGTTTCCCGACTGGAAAGCGGGCAGTGAGCGCAACGCAATTAATGTGAGTTAGCTCACTCATTAGGCACCCCAGGCTTTACACTTTATGCTTCCGGCTCGTATGTTGTGTGGAATTG
TGAGCGGATAACAATTTCACAGTTTAAACCAGGAAACAGCTATGACCATGGGATCCGAACAAAAACTCATCTCAGAAGAGGATCTGATGAATTCAGGCGGCGGAGGGACGTCCCCGGGACTAGTCTACGTACGGCAGTTTAAGGTTTACACCTATAAAAGAGAGAGCCGTTATCGTCTGTTTGTG
GATGTACAGAGTGATATTATTGACACGCCGGGGCGACGGATGGTGATCCCCCTGGCCAGTGCACGTCTGCTGTCAGATAAAGTCTCCCGTGAACTTTACCCGGTGGTGCATATCGGGGATGAAAGCTGGCGCATGATGACCACCGATATGGCCAGTGTGCCGGTCTCCGTTATCGGGGAAGAAGTG
GCTGATCTCAGCCACCGCGAAAATGACATCAAAAACGCCATTAACCTGATGTTCTGGGGAATATAAACTAGTGTCGGGGTCGGGGTCGACTTTCTCGAGATTGAACAAGATGGATTGCACGCAGGTTCTCCGGCCGCTTGGGTGGAGAGGCTATTCGGCTATGACTGGGCACAACAGACAATCGG
CTGCTCTGATGCCGCCGTGTTCCGGCTGTCAGCGCAGGGGCGCCCGGTTCTTTTTGTCAAGACCGACCTGTCCGGTGCCCTGAATGAACTGCAAGACGAGGCAGCGCGGCTATCGTGGCTGGCCACGACGGGCGTTCCTTGCGCAGCTGTGCTCGACGTTGTCACTGAAGCGGGAAGGGACTG
GCTGCTATTGGGCGAAGTGCCGGGGCAGGATCTCCTGTCATCTCACCTTGCTCCTGCCGAGAAAGTATCCATCATGGCTGATGCAATGCGGCGGCTGCATACGCTTGATCCGGCTACCTGCCCATTCGACCACCAAGCGAAACATCGCATCGAGCGAGCACGTACTCGGATGGAAGCCGGTCTTG
TCGATCAGGATGATCTGGACGAAGAGCATCAGGGGCTCGCGCCAGCCGAACTGTTCGCCAGGCTCAAGGCGAGCATGCCCGACGGCGAGGATCTCGTCGTGACCCATGGCGATGCCTGCTTGCCGAATATCATGGTGGAAAATGGCCGCTTTTCTGGATTCATCGACTGTGGCCGGCTGGGTGT
GGCGGACCGCTATCAGGACATAGCGTTGGCTACCCGTGATATTGCTGAAGAGCTTGGCGGCGAATGGGCTGACCGCTTCCTCGTGCTTTACGGTATCGCCGCTCCCGATTCGCAGCGCATCGCCTTCTATCGCCTTCTTGACGAGTTCTTCTGACTCGAGGGGATCGGGCCCTACGTACGGCAGTT
TAAGGTTTACACCTATAAAAGAGAGAGCCGTTATCGTCTGTTTGTGGATGTACAGAGTGATATTATTGACACGCCGGGGCGACGGATGGTGATCCCCCTGGCCAGTGCACGTCTGCTGTCAGATAAAGTCTCCCGTGAACTTTACCCGGTGGTGCATATCGGGGATGAAAGCTGGCGCATGATGAC
CACCGATATGGCCAGTGTGCCGGTCTCCGTTATCGGGGAAGAAGTGGCTGATCTCAGCCACCGCGAAAATGACATCAAAAACGCCATTAACCTGATGTTCTGGGGAATATAAATGTCAGGCCTGAATGTCTAGAGAGCTTGGCTGTTTTGGCGGATGAGAGAAGATTTTCAGCCTGATACAGATTA
AATCAGAACGCAGAAGCGGTCTGATAAAACAGAATTTGCCTGGCGGCAGTAGCGCGGTGGTCCCACCTGACCCCATGCCGAACTCAGAAGTGAAACGCCGTAGCGCCGATGGTAGTGTGGGGTCCCCCATGCGAGAGTAGGGAACTGCCAGGCATCAAATAAAACGAAAGGCTCAGTGCAAAG
ACTGGGCCTTTCGTTTTATCTGTTGTTTGTCGGTGAACGCTCTCCTGAGTAGGACAAATCCGCCGGGAGCGGATTTGAACGTTGCGAAGCAACGGCCCGGAGGGTGGCGGGCAGGACGCCCGCCATAAACTGCCAGGCATCAAATTAAGCAGAAGGCCATCCTGACGGATGGCCTTTTTGCGTT
TCTACAAACTCTTTGTTTATTTTTCTAAATACATTCAAATATGTATCCGCTCATGAGACAATAACCCTGATAAATGCTTCAATAATATTGAAAAAGGAAGAGTATGAGTATTCAACATTTCCGTGTCGCCCTTATTCCCTTTTTTGCGGCATTTTGCCTTCCTGTTTTTGCTCACCCAGAAACGCTGGT
GAAAGTAAAAGATGCTGAAGATCAGTTGGGTGCACGAGTGGGTTACATCGAACTGGATCTCAACAGCGGTAAGATCCTTGAGAGTTTTCGCCCCGAAGAACGTTTTCCAATGATGAGCACTTTTAAAGTTCTGCTATGTGGCGCGGTATTATCCCGTGTTGACGCCGGGCAAGAGCAACTCGGTC
GCCGCATACACTATTCTCAGAATGACTTGGTTGAGTACTCACCAGTCACAGAAAAGCATCTTACGGATGGCATGACAGTAAGAGAATTATGCAGTGCTGCCATAACCATGAGTGATAACACTGCGGCCAACTTACTTCTGACAACGATCGGAGGACCGAAGGAGCTAACCGCTTTTTTGCACAAC
ATGGGGGATCATGTAACTCGCCTTGATCGTTGGGAACCGGAGCTGAATGAAGCCATACCAAACGACGAGCGTGACACCACGATGCCTGCAGCAATGGCAACAACGTTGCGCAAACTATTAACTGGCGAACTACTTACTCTAGCTTCCCGGCAACAATTAATAGACTGGATGGAGGCGGATAAAGT
TGCAGGACCACTTCTGCGCTCGGCCCTTCCGGCTGGCTGGTTTATTGCTGATAAATCTGGAGCCGGTGAGCGTGGGTCTCGCGGTATCATTGCAGCACTGGGGCCAGATGGTAAGCCCTCCCGTATCGTAGTTATCTACACGACGGGGAGTCAGGCAACTATGGATGAACGAAATAGACAGATCGC
TGAGATAGGTGCCTCACTGATTAAGCATTGGTAACTGTCAGACCAAGTTTACTCATATATACTTTAGATTGATTTACGCGCCCTGTAGCGGCGCATTAAGCGCGGCGGGTGTGGTGGTTACGCGCAGCGTGACCGCTACACTTGCCAGCGCCCTAGCGCCCGCTCCTTTCGCTTTCTTCCCTTCCTT
TCTCGCCACGTTCGCCGGCTTTCCCCGTCAAGCTCTAAATCGGGGGCTCCCTTTAGGGTTCCGATTTAGTGCTTTACGGCACCTCGACCCCAAAAAACTTGATTTGGGTGATGGTTCACGTAGTGGGCCATCGCCCTGATAGACGGTTTTTCGCCCTTTGACGTTGGAGTCCACGTTCTTTAATAG
TGGACTCTTGTTCCAAACTGGAACAACACTCAACCCTATCTCGGGCTATTCTTTTGATTTATAAGGGATTTTGCCGATTTCGGCCTATTGGTTAAAAAATGAGCTGATTTAACAAAAATTTAACGCGAATTTTAACAAAATATTAACGTTTACAATTTAAAAGGATCTAGGTGAAGATCCTTTTTGATA
ATCTCATGACCAAAATCCCTTAACGTGAGTTTTCGTTCCACTGAGCGTCAGACCCCGTAGAAAAGATCAAAGGATCTTCTTGAGATCCTTTTTTTCTGCGCGTAATCTGCTGCTTGCAAACAAAAAAACCACCGCTACCAGCGGTGGTTTGTTTGCCGGATCAAGAGCTACCAACTCTTTTTCCG
AAGGTAACTGGCTTCAGCAGAGCGCAGATACCAAATACTGTCCTTCTAGTGTAGCCGTAGTTAGGCCACCACTTCAAGAACTCTGTAGCACCGCCTACATACCTCGCTCTGCTAATCCTGTTACCAGTGGCTGCTGCCAGTGGCGATAAGTCGTGTCTTACCGGGTTGGACTCAAGACGATAGTTA
CCGGATAAGGCGCAGCGGTCGGGCTGAACGGGGGGTTCGTGCACACAGCCCAGCTTGGAGCGAACGACCTACACCGAACTGAGATACCTACAGCGTGAGCATTGAGAAAGCGCCACGCTTCCCGAAGGGAGAAAGGCGGACAGGTATCCGGTAAGCGGCAGGGTCGGAACAGGAGAGCGCAC
GAGGGAGCTTCCAGGGGGAAACGCCTGGTATCTTTATAGTCCTGTCGGGTTTCGCCACCTCTGACTTGAGCGTCGATTTTTGTGATGCTCGTCAGGGGGGCGGAGCCTATGGAAAAACGCCAGCAACGCGGCCTTTTTACGGTTCCTGGCCTTTTGCTGGCCTTTTGCTCACATGTTCTTTCCTGC
GTTATCCCCTGATTCTGTGGATAACCGTATTACCGCCTTTGAGTGAGCTGATACCGCTCGCCGCAGCCGAACGACCGAGCGCAGCGAGTCAGTGAGCGAGGAAGCGGAAGAGCGCCTGATGCGGTATTTTCTCCTTACGCATCTGTGCGGTATTTCACACCGCATATGGTGCACTCTCAGTACAAT
CTGCTCTGATGCCGCATAGTTAAGCCAGTATACACTCCGCTATCGCTACGTGACTGGGTCATGGCTGCGCCCCGACACCCGCCAACACCCGCTGACGCGCCCTGACGGGCTTGTCTGCTCCCGGCATCCGCTTACAGACAAGCTGTGACCGTCTCCGGGAGCTGCATGTGTCAGAGGTTTTCAC
CGTCATCACCGAAACGCGCGAGGCAGCAAGGAGATGGCGCCCAACAGTCCCCCGGCCACGGGGCCTGCCACCATACCCACGCCGAAACAAGCGCTCATGAGCCCGAAGTGGCGAGCCCGATCTTCCCCATCGGTGATGTCGGCGATATAGGCGCCAGCAACCGCACCTGTGGCGCCGGTGAT
GCCGGCCACGATGCGTCCGGCGTAGAGGATCTAATTCTCATGTTTGACAGCTTATC

Nucleotide sequence of the backbone plasmid (ptMAX), which was prepared for pdGENE-Toxin sensitivity assay. This plasmid contains three CcdB genes (one CcdB gene in arabinose unit and two CcdB genes in IPTG unit).

Supplementary Information Figure 6

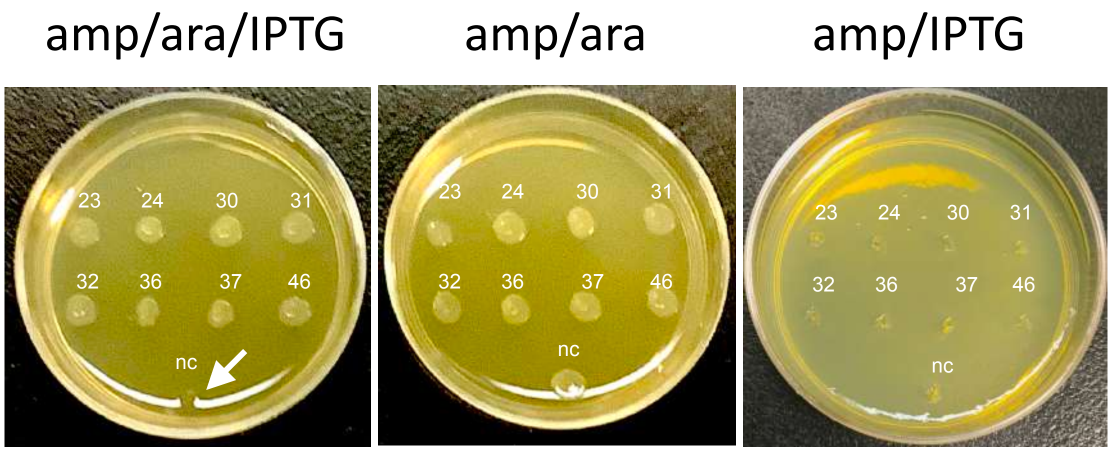

nc: clone #2, which is IPTG sensitive.

Results of third screening of pdGENE-Toxin sensitivity assay.

(Candidate clones with selection in Figure 3)

Selection by ampicillin, arabinose, and IPTG (left), ampicillin and arabinose (middle), and ampicillin and IPTG (right). #23, #24, #30, #31, #32, #36, #37 and #46 form colonies under ampicillin/arabinose/IPTG, ampicillin/arabinose, and ampicillin/IPTG. Negative control was clone #2, which was IPTG-sensitive (negative selection). Without arabinose induction, all clones were IPTG-sensitive (negative selection).

Supplementary Information Figure 7
